# Comparative Analysis of the Cell Fates of Induced Schwann Cells from Subcutaneous Fat Tissue and Naïve Schwann Cells in the Sciatic Nerve Injury Model

**DOI:** 10.1155/2017/1252851

**Published:** 2017-06-20

**Authors:** Mingzi Zhang, Mei Hua Jiang, Dae-Wook Kim, Woosung Ahn, Eunkyung Chung, Youngsook Son, Guangfan Chi

**Affiliations:** ^1^Department of Genetic Engineering, College of Life Science and Graduate School of Biotechnology, Kyung Hee University, Seocheon-dong, Giheung-gu, Yongin-si 446-701, Republic of Korea; ^2^Department of Pathobiology, The Key Lab of Educational Ministry, Jilin University, Changchun, China; ^3^Department of Anatomy and Neurobiology, Zhongshan School of Medicine, Sun Yat-sen University, Guangzhou, China

## Abstract

**Purpose:**

The fate and function of the induced Schwann cells (iSCs) like cells from adipose tissue have not been critically evaluated in vivo after transplantation. The objective of this study is to compare the fate of iSCs with naïve SCs (nSCs) after transplantation into the lesion sites of sciatic nerve, respectively.

**Methods:**

Adipose-derived stem cells from eGFP-expressing transgenic rat's subcutaneous fat were induced to iSCs in vitro. iSCs were injected to the sciatic nerve lesion area after crush injury and the cells fate was comparatively analyzed with that of nSCs from the same rat.

**Results:**

At 12 weeks after transplantation, nSCs were detected only in the restricted area of cell transplantation site but iSCs were widely distributed all over the sciatic nerve. Based on double fluorescence observations, both iSCs and naïve ones were colocalized with P0-expressing myelin sheath, outbound by laminin-expressing basal membrane, and terminated at contactin-associated protein-expressing doublets. However, some of iSCs were also differentiated to the fibrocyte/fibroblast-like cells. In the histological analysis of repaired sciatic nerves, axon density was higher in iSC-received group than in the nSCs group and normal sciatic nerve.

**Conclusion:**

iSCs induced from subcutaneous fat tissues have higher engraftment and migration capacity than nSCs.

## 1. Introduction

Schwann cells (SCs) are a major component of the peripheral nervous system (PNS), which myelinate axons, aid in the directional guidance of neurons, and eliminate cellular debris [1]. SCs are known to secrete various neurotrophic factors, such as nerve growth factor (NGF), brain-derived neurotrophic factor (BDNF), glial cell line-derived neurotrophic factor (GDNF), and ciliary neurotrophic factor (CNTF); they also produce or secrete extracellular matrix molecules such as laminin [2]. SCs have been proposed for the cell therapy in PNS and central nervous system (CNS) injuries. Previous results have described that SCs promoted axonal regeneration and myelination when transplanted into adult CNS lesions, such as in the optic nerve and the spinal cord [3–5].

SCs may be isolated from autogenic or allogenic sciatic nerve. However, the use of autogenic naïve SCs is limited by their poor accessibility and morbidity at the donor tissue. Furthermore, allogenic SCs are known to be involved in acute or chronic immune reactions. Hence, alternative sources of autogenic SCs or their equivalent other candidates have been expected to be uncovered. The use of olfactory ensheathing cells [6], boundary cap neural crest stem cells [7], skin-derived precursors [8, 9], and bone marrow stromal cells [10, 11] has been attempted for this purpose; however, their accessibility and low yield have posed major obstacles to further research for clinical application.

Adipose tissue is widely available in the body and has been considered as an alternative source of stromal cells capable of differentiating into mesodermal lineages such as osteogenic, adipogenic, chondrogenic, and ectodermal lineages such as neuronal and glial cells [12–14]. Moreover, the frequency of adult stem cells in adipose tissue is higher than that in bone marrow [15–18]. Previously, several investigations reported that adult stem cells from adipose tissue can differentiate into SCs and promote neurite outgrowth in vitro [19–21]. In our previous report, we have found that spheroids derived from adipose tissue could efficiently differentiate into SC-like cells in vitro and exhibited SCs traits in the spinal cord injured rat model [22]. In this study, we used the same protocol to isolate spheroid-forming cells from subcutaneous adipose tissue of eGFP-expressing transgenic rats and induced into a SC phenotype in vitro. Then, to assess their functional equivalence to nSCs in the repair of damaged peripheral nerve tissue, we comparatively analyzed their engraftment and myelination in a sciatic nerve crush injury model.

## 2. Materials and Methods

### 2.1. Isolation of Subcutaneous Tissue Cells and Culture of Spheres

All animal experiments were approved by the Institutional Animal Care and Use Committee of Kyung Hee University. Adult male Sprague-Dawley rats (eGFP transgenic rat) (9~10 weeks old, Japan SLC, Hamamatsu, Japan) were anaesthetized, and subcutaneous tissue was carefully dissected under aseptic conditions. This tissue was washed with phosphate-buffered saline (PBS) (WelGENE Inc., Daegu, Korea) comprising 100 U/mL penicillin and 100 *μ*g/mL streptomycin (Invitrogen, Carlsbad, CA) to remove the contaminated debris and red blood cells. Then, the tissue was mechanically minced to ~1 mm^3^ by using a surgical scissor, treated with 0.15% collagenase type I (Worthington Biochemical Corporation, Lakewood, NJ), and left to stand for 1 h at 37°C. The tissue was treated with Dulbecco's modified Eagle's medium/F12 (DMEM/F12) (3 : 1) (WelGENE Inc.) comprising 10% fetal bovine serum (FBS; Lonza, Walkersville, MD) at the same volume as collagenase in order to inactivate the collagenase and centrifuged at 1500 rpm/min for 5 min. The supernatant was carefully discarded, and the remainder was washed with DMEM/F12 (3 : 1) twice and then filtered through a 40 *μ*m strainer (BD Biosciences, Mississauga, Ontario) to remove debris. The filtrate was centrifuged, and the pellet was resuspended in DMEM/F12 (3 : 1) medium comprising N2 supplement (Invitrogen), 20 ng/mL EGF (R&D Systems Inc., Minneapolis, MN), 20 ng/mL bFGF (R&D), and 2 *μ*g/mL heparin (Sigma-Aldrich, St. Louis, Missouri) and plated in a 75 cm^2^ flask (Nunc, Roskilde). The medium was refreshed every 3 d. After 7~10 d, the spheres formed therein were isolated by centrifuging the culture medium and dissociated to single cells by using a fire-polished Pasteur pipette (Corning, Austin, Texas). Such dissociated cells were spun down and resuspended in DMEM/F12 (3 : 1) comprising 10% FBS and plated in a 75 cm^2^ flask at 5 × 10^5^/mL density. Two-thirds (2/3) of the medium were replaced every 3 d, and the cells were separated to single cells by using 0.25% trypsin-EDTA (Invitrogen) at 90% confluence. These cells were diluted in a ratio of 1 : 3, plated to a 75 cm^2^ flask, and subcultured to Passage 5. The subcultured cells were cultured in DMEM/F12 (3 : 1) comprising 20 ng/mL bFGF, 20 ng/mL EGF, N2 supplement, 2 *μ*g/mL heparin, and 1% penicillin/streptomycin for 3 d. The medium was then completely changed to a Neurobasal Medium (Invitrogen) comprising G5 supplement (Invitrogen), 2 *μ*g/mL laminin (Sigma), and 2 *μ*g/mL heparin and the cells were cultured therein for 3-4 d.

### 2.2. Differentiation Induction to SC Phenotype

The spheres cultured in the G5-containing medium (Invitrogen) were centrifuged and plated onto a poly-L-lysine-coated dish (Sigma) and cultured in alpha-minimum essential medium (*α*-MEM) (Invitrogen) comprising 10% FBS, 35 ng/mL all-trans retinoic acid (Sigma), 100 *μ*g/mL penicillin, and 100 *μ*g/mL streptomycin for 4 d. After 4 d of culture, the cells were separated by using 0.25% trypsin-EDTA solution, plated onto a poly-L-lysine-coated dish, and cultured in *α*-MEM (Invitrogen) comprising 10% FBS, 5 *μ*M forskolin, 10 ng/mL bFGF, 5 ng/mL platelet-derived growth factor antibodies (PDGF-AA) (R&D), and 200 ng/mL heregulin1-*β*1 for 8 d, and 2/3 of the medium were refreshed every 4 d. After confluence to 80%, the cells were separated and subcultured for 20 d in the same medium with the addition of 10 ng/mL NT3 (neurotrophin-3) (R&D).

### 2.3. Immunofluorescence Staining

The cells induced to SC-like cells were differentiated, subcultured, seeded on cover slips, and cultured for 4 d in *α*-MEM comprising 10% FBS, 5 *μ*M forskolin, 10 ng/mL bFGF, 5 ng/mL PDGF-AA, 200 ng/mL heregulin1-*β*1, and 10 ng/mL NT3; then, immunofluorescence analysis was performed. The cells were fixed with a solution of 4% (v/v) paraformaldehyde (PFA, Sigma) in 0.1 M PBS (pH 7.4) for 20 min. After washing with PBS 3 times each for 10 min, nonspecific antigens were blocked with 10% goat serum (Vector Laboratories, Burlingame, CA) in PBS for 1 h and incubated with the primary antibodies overnight at 4°C. For S100*β* staining, the cells on the some cover slips were additionally permeabilized in 0.2% Triton X-100 (USB Corporation, Cleveland, OH) in PBS before blocking with goat serum as mentioned above. The dilutions used were as follows: mouse anti-O4 antibody (Chemicon, Temecula, CA; MAB345, 1 : 500) and mouse anti-A2B5 antibody and rabbit anti-S100*β* antibody (Dako, Carpinteria, CA; Z0311, 1 : 200). Finally, the cells were washed with PBS 3 times and incubated with anti-mouse IgG (Vector Laboratories; TI-2000, 1 : 200) or anti-rabbit IgG (Vector Laboratories; TI-1000, 1 : 200) Texas-red-conjugated secondary antibody at room temperature for 1 h. After washing 3 times in PBS, cover slips were mounted onto slides with VECTASHIELD Mounting Medium (Vector Laboratories) containing 4,6-diamidino-2-phenylindole (DAPI). The results were examined using a Leica fluorescence microscope (Leica, Solms, Germany).

### 2.4. Adult SCs Culture

The primary culture of rat adult SCs was performed according to previous reports with minor modifications [22]. Briefly, the sciatic nerves were dissected from the hind limbs of adult male eGFP transgenic rats (7 weeks, Japan SLC). The epineuria, connective tissue, and blood vessels were stripped off from the sciatic nerves with fine forceps and minced into 1 × 1 mm explants with a microscissor under an autopsy microscope (Olympus, Tokyo, Japan). These explants were cultured in a 60 mm culture dish with 4-5 mL of DMEM/F12 (3 : 1) containing 10% FBS (v/v) at 37°C in 5% CO_2_. In order to prevent the floating of the explants, fresh medium was replenished once a week until the outgrowth of fibroblast reached a confluent monolayer around the explants. The explants were then moved to a new culture dish. This procedure was repeated until fibroblast migration from the explants ceased and bipolar SCs migration increased; this generally needed 3-4 weeks. The explants were mixed with 0.25% trypsin-EDTA by gently triturating through Pasteur pipettes and incubated for 10 min in a 37°C water bath. The cells were mixed with *α*-MEM containing 10% FBS, replated on the poly-L-lysine-coated dish after centrifugation, and cultured in *α*-MEM containing 10% FBS, 2 *μ*m forskolin, and 50 ng/mL heregulin1-*β*1. The medium was exchanged once a week and cells were subcultured to Passage 3. Based on the cell morphology and S100*β* antigen immunostaining, the purity of the SC culture was approximately more than 90%.

### 2.5. Reverse Transcription-Polymerase Chain Reaction (RT-PCR)

The cells at each culture stage were separated by 0.25% trypsin-EDTA, washed, and centrifuged in PBS containing 5% serum at 1500 rpm/min for 5 min. After washing and centrifuging in PBS twice, mRNA was prepared from samples by using the RNase mini kit (Qiagen, Valencia, CA), and cDNA was generated with Superscript™ III first strand synthesis system (Invitrogen) for PCR, according to the manufacturer's protocol. PCR was performed with 100 ng cDNA by using Perfect PreMix ver. 2.1 (TaKaRa, Otsu, Shiga). PCR reactions were normalized by amplifying the same cDNA samples with primers for GAPDH (glyceraldehyde-3-phosphate dehydrogenase). Detailed primers sequences and annealing temperatures are given in Table 1.

### 2.6. Sciatic Nerve Injury and Cell Transplantation

Prior to grafting the cells, the differentiated cells from Passage 5 and normal SCs were treated with 0.25% trypsin-EDTA, detached individually from cell culture flasks, and resuspended in DMEM/F12 (3 : 1) medium containing 10% FBS. After washing and centrifuging twice at 1500 rpm/min for 5 min, these cells were diluted to the density of 5 × 10^7^/mL in PBS and placed at 4°C in an ice box until transplantation. The cells viability was confirmed to be more than 95% by Trypan blue exclusion after labeling.

Adult female Sprague-Dawley rats (weighing 250–300 g, *n* = 20) were divided into two groups as follows:

1. Group A: transplantation of iSCs (*n* = 10)
2. Group B: transplantation of nSCs (*n* = 10)

The animals were anaesthetized by intraperitoneal administration of ketamine (80 mg/kg) (Yuhan, Seoul, Korea) and Rompun (7.4 mg/kg) (Bayer, Seoul, Korea). The hair over the right femur was removed and the area was sterilized with 10% povidone iodine. The right thigh's sciatic nerve was exposed by opening the fascial plane between the gluteal musculature and femoral musculature via a longitudinal incision and dissected free from the surrounding connective tissue. The crush injury was performed by fully pressing a 2-3 mm segment of sciatic nerve at mid-thigh level for 30 s with a forceps (Dumont #5) (WPI, Sarasota, FL). Immediately after injury, the prepared cell mixture (1 *μ*L of 5 × 10^7^/mL,) was directly injected into the crush site using a 5 *μ*L Hamilton syringe attached to a 30-gauge needle. Then, both the rostral and caudal margins of the lesion site were marked at the epineurium with 10-0 nondegradable sutures individually for future identification. The muscle and skin were sutured subsequently with 6-0 sutures. After the operation, these rats were kept on heating pads, observed until fully awake, and returned to their cages. In the control group, the same number of nSCs was injected and the rest of procedures were identical to those mentioned above. Cefazolin (Chong Kun Dang, Seoul, Korea) was administered by intraperitoneal injection for 1 week. All of the rats underwent immunosuppression with a single daily injection of cyclosporine (Chong Kun Dang, Seoul, Korea) administered subcutaneously at a dose of 1 mg/100 g starting 3 d before the transplantation and continued to the end time of experiment.

### 2.7. Tissue Preparation

At the end of 12 weeks, the rats were sacrificed, respectively. The sciatic nerves were dissected out, immersed in 4% paraformaldehyde, and maintained at 4°C for 24 h. Then, these samples were immersed in 30% sucrose (Sigma) for 3 d. The lesion site was identified and horizontally or vertically blocked in OCT compound (Sakura, Chuo-ku, Tokyo) and maintained at –80°C until examination.

### 2.8. Histology and Immunofluorescence Staining

The samples were sectioned at 20 *μ*m thickness by crystal sectioning and mounted onto Superfrost Plus Slides (VWR, Lutterworth, Leicestershire). For histological examination, frozen sections were dried completely and placed in PBS for 10 min and mounted with VECTASHIELD mounting medium containing DAPI. For immunofluorescence analysis, the sections were immersed in 0.3% Triton X-100 in PBS for 1 h. After washing in PBS 3 times, these sections were blocked with 10% normal goat serum or 10% horse serum for 1 h at room temperature. The following primary antibodies were applied to these sections and incubated at 4°C overnight: goat-anti P0 (Santa Cruz; SC-18531, 1 : 50), rabbit-anti laminin (Dako; Z0097, 1 : 50), and rabbit-anti contactin-associated protein (Caspr) (Abcam, Cambridge, MA; ab34151, 1 : 150). The following day, these sections were washed 3 times in PBS and then reacted with Tex-red-conjugated secondary antibodies (Vector Laboratories; rabbit anti-goat TI-5000 or goat anti-rabbit TI-1000, 1 : 200) for 1 h at room temperature in a humidified chamber. After washing 3 times in PBS, these sections were cover-slipped with VECTASHIELD mounting medium containing DAPI. The sections were observed under a fluorescence microscope (Leica CTR 4000), and digital images were captured with a Zeiss LSM 510 META confocal microscope (Carl Zeiss, Jena, Germany).

### 2.9. Quantitative Analysis of Repaired Tissue and Transmission Electron Microscopic Analysis

At end time of 12 weeks, the incised sciatic nerve segments from remainder rats were fixed in 2.5% glutaraldehyde buffered in cacodylate (0.025 M), post-fixed for 1.5 h in 2% osmium tetroxide, washed in PBS, and dehydrated through a graded ethanol series. For transversal section at central area within lesion site, these samples were just transversely cut off at middle site and vertically embedded in epon. Transverse semithin sections at 1 *μ*m thickness were made and stained with toluidine blue. Five randomly selected representative fields of known areas (100 *μ*m × 100 *μ*m) were evaluated in per nerve section. In each field, the numbers of axons were counted and the axons diameters including myelin sheath thickness and myelin sheath thickness individually were measured using an image analysis system at ×640 magnification of light microscope. Finally, these data were statistically analyzed.

The ultrathin sections (70 nm thick) were observed under transmission electron microscope and the representative microphotos were taken at ×4400 magnification.

### 2.10. Statistical Analysis

Data were presented as mean values ± standard deviation, and Student's* t-*tests were applied as appropriate. The statistical significance of differences in parameters was accepted at *P* < 0.05.

## 3. Results

### 3.1. Induction of Nestin-Expressing Spheroids with Proliferating Capacity from the Subcutaneous Fat Tissue of eGFP Transgenic Rat

**Figure 1:**
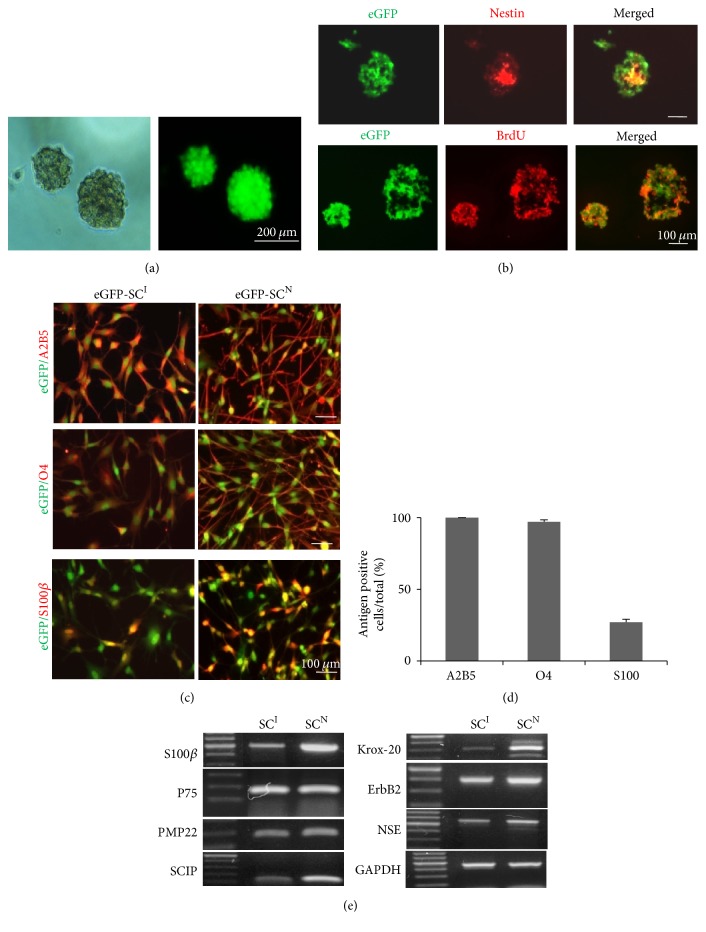
Induction of spheroids from subcutaneous fat tissue of eGFP transgenic rat and their SCs differentiation. (a) Efficient induction of spheroids from adipose cells of eGFP transgenic rats. Upon exposure to the induction medium containing bFGF and EGF, adherent adipose tissue derived cells were merged to the spheroid and free floating ball-like structures were formed approximately at 6 d. (b) Characterization of the spheroid by immunofluorescence staining with anti-nestin antibody and BrdU labeling. Nestin-immunoreactive cells were negative at the periphery of the spheroid but confined to the inner part of the spheroid at 8 d induction. Most cells in the spheroid showed cell-proliferating capacity. (c) Immunofluorescence staining with anti-O4, anti-A2B4, and anti-S100*β* antibodies to confirm glial differentiation of the spheroid-forming cells in comparison with nSCs isolated from normal sciatic nerve. (d) Quantification of A2B5, O4, and S100*β* positive cells among total eGFP-expressing cells after immunofluorescence staining. (e) RT-PCR analysis for SCs specific genes expression. Both eGFP-SC^I^ and eGFP-SC^N^ exhibited similar gene expression profiles. eGFP-SCs^I^: induced Schwann cells of eGFP transgenic rat; eGFP-SCs^N^: naïve Schwann cells of eGFP transgenic rat.

Neurospheroid-like structures have been previously identified in newborn rat, mouse skin, and human foreskin [23–26] and also adult subcutaneous fat tissue devoid of hair follicles and other skin appendages, which were efficiently induced to SC-like cells and engaged in the SCI [22]. In order to investigate the fate of induced SC-like cells in the repair of PNS in comparison with nSCs, SC-like cells were induced from the spheroid-forming cells derived from the subcutaneous fat tissue of eGFP-expressing transgenic rats according to our previous report [22] and nSCs were isolated from the sciatic nerve of eGFP-transgenic rats. Spheroids began to form at day 3 and grew to 100–200 *μ*m in diameter at day 10 (Figure 1(a)). Approximately 60–80% of the initially adherent cells generated the eGFP-expressing spheroid after exposure to the induction medium.

Then, to examine the nestin expression and proliferation capacity of the spheroid, whole mount examination of the spheroid was performed after immunofluorescence staining to nestin and BrdU uptake tests (Figure 1(b)). Only the cells centrally located in the spheroid were positive in the nestin expression but the cells in the outer layer were negative in the nestin expression. However, the spheroid was composed almost entirely of BrdU-positive cells. Thus, all the cells in spheroids were able to proliferate but their nestin expression was affected by the spatial positions within the spheroid.

### 3.2. Differentiation of Spheroid-Forming Cells into SC-Like Cells

The spheroid-forming cells were tested for differentiation into SC-like cells using an SC-induction medium. After induction, the cells were elongated with two or three processes. By immunofluorescence staining with antigens against O4, A2B5, and S100*β* (Figures 1(c) and 1(d)), after the SC differentiation, nearly 100% of the cells expressed O4 and A2B5 but only 26% of cells expressed S100*β*, all of which are considered as molecular markers for immature and mature SCs, respectively. In contrast, nSCs were in a much sharper bipolar morphology, which were approximately twice more elongated than iSCs (data not shown). All the nSCs expressed very strongly O4, A2B5, and S100*β* even though some were negative in S100*β* expression.

RT-PCR analyses of the differentiated cells and nSCs were compared (Figure 1(e)). The differentiated cells constantly expressed SC markers such as p75, S100*β*, PMP22, Krox-20, SCIP, ErbB2, and NSE. These gene expression patterns were similar to those of nSCs. Here, such induced SCs were named as eGFP-SCs^I^ in contrast to naïve SC (eGFP-SCs^N^).

### 3.3. Higher Engraftment and Longer Migration of eGFP-SCs^I^ than eGFP-SCs^N^ after Transplantation in Sciatic Nerve Crush Injury

**Figure 2:**
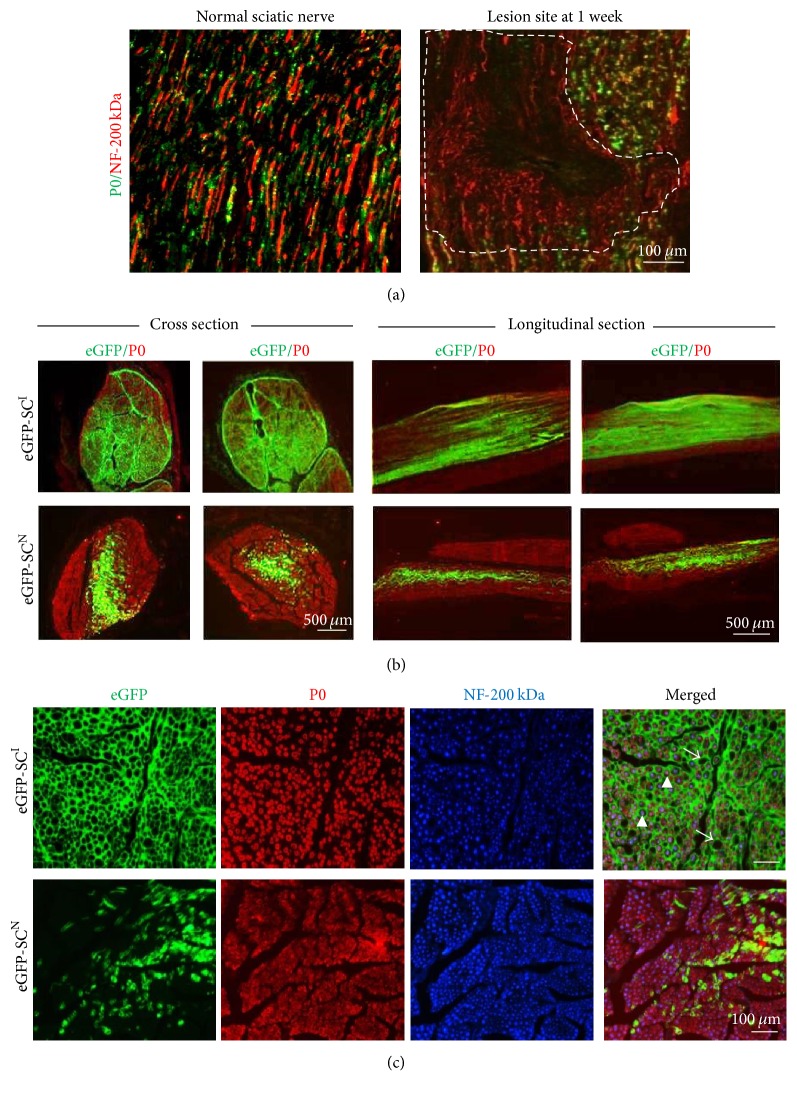
Histological analysis of the sciatic nerve injured site. (a) After crush injury of sciatic nerve at 1 week, the neurofilament 200 kDa (NF-200 kDa) expressing nerve axons were completely distorted and P0-expressing SCs disappeared in the lesion site indicated by dotted line. (b) Fluorescence images of eGFP-expressing cells (green) and immunofluorescence staining with anti-P0 (red) antibodies in cross sections and longitudinal sections of sciatic injury area. In the eGFP-SCs^I^ transplanted group, GFP fluorescence was more widely and evenly detected in most areas of the injured site; however, in the eGFP-SCs^N^ transplanted group, GFP fluorescence was mainly concentrated at the injection site and migrated along the longitudinal lines of the axon. Although identical numbers of cells were transplanted, more cells were detected in the wider area of the injured site. P0 proteins were evenly observed in all areas of the cell-injected sites in both eGFP-SCs^I^ and eGFP-SCs^N^ groups, indicating myelin sheath formation in the entire area of the injured site. (c) Fluorescence images of GFP + cells (green) and double immunofluorescence staining with anti-P0 (red) antibodies and NF-200 (blue) antibodies in cross sections of sciatic injury area. In the eGFP-SCs^I^ transplanted group, the eGFP-expressing cells were connected to each other and formed honeycomb-like structures, which often have empty holes. P0-expressing ring-like myelin sheath was tightly wrapping one of NF-200 positive axons (arrowheads). Some of GFP-expressing holes were empty, which are supposed to be a vascular structure (arrows). On the contrary, eGFP-SCs^N^ were sparsely distributed and not interconnected to others. Each of eGFP-SCs^N^ wrapped up an axon and was tightly coincided with P0-positive myelin sheath.

Crush injury to the sciatic nerve results in the intact epineuria and disruption of SCs, axons, and other cellular structures within the injured side. At day 7 after injury, the lesion cavity deficient in axon and myelin sheath was obvious (Figure 2(a)). Numerous denuded NF-200 positive axons lacking in P0-expressing myelin sheath were growing into the lesion and disorganized P0-expressing SCs were abundant at the lesion periphery. Therefore, this injury model is suitable for evaluating whether the transplanted cells retain SC traits in vivo or not as well as for comparative analysis of engraftment rates between eGFP-SCs^I^ and eGFP-SCs^N^.

After sciatic nerve injury and cells transplantation, the injured hind limbs showed paralysis for approximately one week and gradually improved to achieve a normal state at twelve weeks. Further, there was no behavioral recovery difference between two groups during the experimental periods. At 12 weeks, no abnormal outgrowth of tissue or inflammatory reaction was detected.

At low magnification of 12 weeks' longitudinal and cross sections (Figure 2(b)), the injured sites were fully restored with P0-expressing SCs in both groups. Transplanted eGFP-SCs^I^ were distributed more evenly to the whole injury site, much longer along the longitudinal line and wider than eGFP-SCs^N^. Transplanted eGFP-SCs^N^ were mainly confined in the central injection area of the injury site. This indicates that eGFP-SCs^I^ have more active migration capability than eGFP-SCs^N^ in in vivo environment. Thus, the engraftment of transplanted cells was much higher in eGFP-SCs^I^ transplanted group than in the eGFP-SCs^N^ transplanted group.

To analyze the fate of eGFP-SCs^N^ and eGFP-SCs^I^ after the transplantation, triple fluorescence observations after double immunofluorescence staining to NF-200 for axon and P0 for myelin sheath were performed in the cross sections (Figure 2(c)). In eGFP-SCs^N^ transplanted group, all the NF-200-positive axons were tightly wrapped either by P0-expressing endogenous SCs (P0-positive but eGFP-negative one) or by P0-expressing transplanted eGFP-SCs^N^. In eGFP-SCs^I^ transplanted group, all the NF-200-positive axons were individually wrapped by P0-expressing myelin sheath and in a rather outward boundary by eGFP-positive cells, which were further interconnected in a network-like structure. In certain area of eGFP-SCs^I^ network-like structure, no axon and no myelin sheath was also detected. Thus, eGFP-SCs^I^ showed the higher cell engraftment and survival after the transplantation than the eGFP-SCs^N^ but their fates to the P0-expressing myelin sheath forming Schwann cells were not strictly guaranteed. Some of them are engaged in the network-like structure, probably representing fibroblast like cells and perineural cells.

### 3.4. Comparative Analysis of Repaired Sciatic Nerve

**Figure 3:**
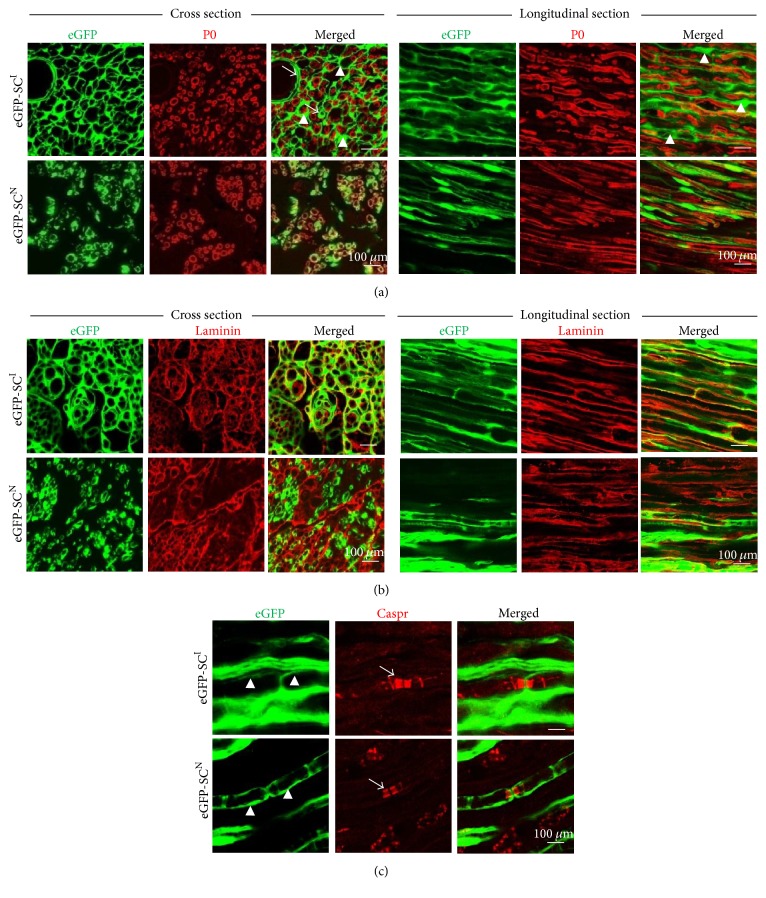
Immunofluorescence stainings for P0, laminin, and Caspr at 12-week transplantation. (a) Dual fluorescence images for GFP (green) and P0 immunofluorescence staining (red). In the eGFP-SCs^I^ group, cytosolic expression of GFP proteins wrapped P0-expressing compact myelin sheath outwardly and segment-like structures of GFP fluorescence were coincided with P0-lacking sites, suggesting nodes of Ranvier structures. Several P0-negative holes were detected, suggesting a vascular structure (arrows) and endoneurium-like structure (arrowheads). In the eGFP-SCs^N^ group, GFP fluorescence also wrapped the P0-expressing hollow tube-like structures outwardly and tightly. (b) Dual fluorescence images for GFP (green) and laminin immunofluorescence staining (red). In the eGFP-SCs^I^ group, laminin immunoreactivities were colocalized with the outer boundary of GFP-expressing cells; segment-like structures of GFP fluorescence were also coincided with laminin immunoreactivities. Colocalization of GFP fluorescence and laminin immunoreactivity was also found in the eGFP-SCs^N^ group. (c) Dual fluorescence images for GFP (green) and Caspr immunofluorescence staining (red, arrows). In the eGFP-SCs^I^ group, doublets of Caspr for myelin sheath at the node of Ranvier were enclosed by two GFP-expressing cells (arrowheads). This symmetric enclosure was also found in the eGFP-SCs^N^ group.

On immunostaining assessment at low magnifications, eGFP-SCs^I^ were distributed in all areas of the lesion but eGFP-SCs^N^ were mostly confined in the central area (Figure 2). At high magnification of cross sections, eGFP-SCs^I^ transplantation showed that a couple of P0-immunoreactive ringlet-like structures were surrounded by single eGFP-positive circular structure, forming an endoneurium-like structure (Figure 3(a)). It was seldom observed that some of eGFP-positive circles did not hold any P0-immunoreactive one, which may represent vascular structure in the sciatic nerve. Some of transplanted eGFP-SCs^I^ were possibly differentiated into fibroblast/fibrocyte and engaged in endoneurium-like structure or perineural cells in lesion site. In contrast, in the eGFP-SCs^N^ group, unlike with eGFP-SCs^I^, almost all eGFP-positive ones were in tight contact with the P0-immunoreactive substance with 1 : 1 relationship and mostly skewed to one side, similarly to normal SC cell body in the myelin sheath. At longitudinal sections, in eGFP-SCs^I^ transplantation, all the P0-immunoreactive tube-like structures were wrapped by eGFP-positive ones. From the relationship between the P0 and eGFP-positive objects, we concluded that the transplanted eGFP-SCs^I^ exhibited SC-like characteristics.

For further detailed verification, we additionally detected the antigen expression of laminin (Figure 3(b)) and Caspr (Figure 3(c)). In normal peripheral nerves, laminin secreted by SCs is a basal lamina component, which surrounds the surface of SCs on the outside. In the eGFP-SCs^I^ and eGFP-SCs^N^ groups, the laminin component was tightly in contact or collocated with the eGFP object on the outside, showing typical basal laminar traits. Caspr is specifically expressed in Ranvier-node structure and is a useful marker to identify the node of Ranvier structures in peripheral nerves. In Caspr antigen analysis, several typical Caspr-positive objects were noted in eGFP-SCs^I^ and eGFP-SCs^N^ groups. Nodes of Ranvier-like structures could be clearly identified between two internodes formed by eGFP-expressing transplanted cells. Caspr-positive objects were located bilaterally to the node of Ranvier-like structures. Also, in the eGFP-SCs^I^ group, thicker eGFP-positive objects which did not participate in forming the nodes of Ranvier-like structures were noted. We suspected that these thicker eGFP-positive objects were formed by fibrocytes/fibroblast that had differentiated from eGFP-SCs^I^. From the immunostaining results, we considered that not all but certain subpopulations of eGFP-SCs^I^ differentiated into SC-like cells and exhibited the functional characteristics of SCs after transplantation but the others differentiated to fibrocytes/fibroblast or periendoneural cells.

**Figure 4:**
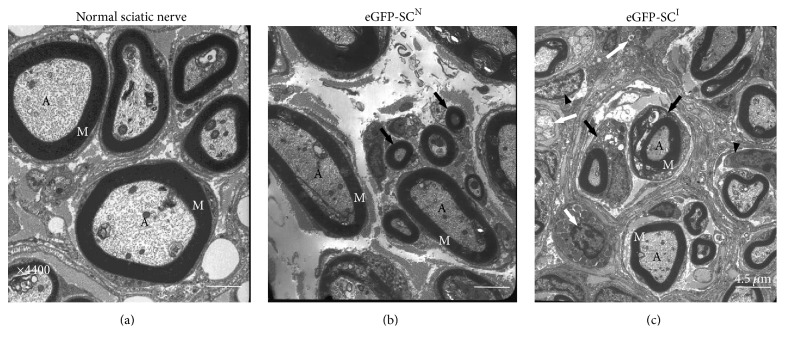
Transmission electron microscopy (TEM) of the injured sciatic nerve at 12-week transplantation. (a) TEM of ultrathin cross section of the normal sciatic nerve. Individual axons were surrounded by dark concentric linings of compact myelin sheaths. (b) TEM of the central zone of the injured site in the eGFP-SCs^N^ transplanted group. Axons were also surrounded by compact myelin sheaths. Unlike in normal controls, axon diameters were fairly diverse. Small and loose myelin sheaths were also noted (arrows). (c) TEM of the central zone of the injured site in the eGFP-SCs^I^ group. Axon diameters and myelin sheath thickness were diverse and smaller than eGFP-SCs^N^ group, and unmyelinating Schwann cells (white arrows) were seldom observed. The space between myelinated nerve fibers was occupied by endoneurium-like cellular structures and fibrocyte/fibroblasts were observed (black arrowheads). A, axons; M, myelin sheath.

Under transmission electron microscopy (Figure 4), normal and eGFP-SCs^N^ groups showed similar morphologic patterns. In these two groups, the myelin sheath-enclosed axons were clearly observed and the basal membrane of SCs was identified. The space among the axons was occupied by extracellular matrix-like collagen fibers along with a few fibrocyte/fibroblast-like cells. In the eGFP-SCs^N^ group, the axons were enclosed by a thick myelin sheath and, unlike normal sciatic nerve, they showed relatively loose myelin sheath structures. In the eGFP-SCs^I^ group, various morphological appearances were noted. In the central area, the axons were enclosed by the myelin sheath but the thickness was thinner than that in the normal sciatic nerve and eGFP-SCs^N^ groups. Occasionally, 2 or 3 axons were surrounded by fibroblast cells and the space between these clusters was occupied by fibrocyte-like cellular structures, which was consistent with immunostaining results. Also, the bigger cell body of wrapping SCs in comparison with those of normal sciatic nerve and eGFP-SCs^N^ group was also noted.

**Figure 5:**
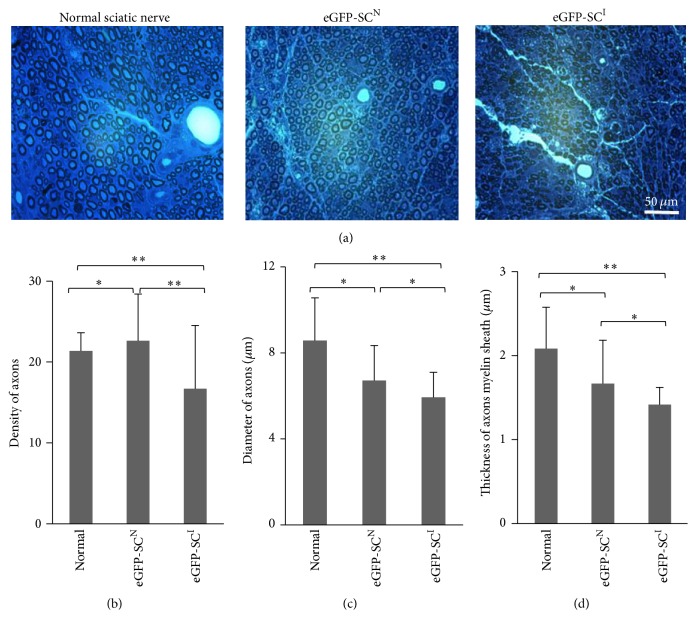
Light micrographs of toluidine blue staining for myelin sheath at the injured area. (a) In normal sciatic nerve, discrete and well-organized perineurial structure was noted and an even distribution of axons within the perineurium was also noted. Axon density was lower in the eGFP-SCs^I^ group than in the eGFP-SCs^N^ group; their diameter was also smaller than that in the normal sciatic nerve. (b–d) Quantitative analysis of axon density (b), axon diameter (c), and thickness of myelin sheath (d) in the 100 *μ*m × 100 *μ*m square area of cross sections. Five images of cross sections of the injury middle line (×640) were analyzed for statistical analysis. Axon density per unit area was lower in normal group (*P* < 0.05). eGFP-SCs^I^ group and eGFP-SCs^N^ showed similar axon density (*P* > 0.05). Axons diameters, including myelin sheath and thickness of myelin sheath, were significantly smaller in the eGFP-SCs^I^ group than in the normal group (*P* < 0.05), and no significant differences with the eGFP-SCs^N^ group were noted (*P* > 0.05). In contrast, eGFP-SCs^N^ did not show any statistical differences with the normal group (*P* > 0.05). Data are represented as mean ± SEM. ^*∗*^*P* > 0.05; ^*∗∗*^*P* < 0.05.

To analyze quantitatively the quality of repaired region, axon density, diameter of axon, and thickness of myelin sheath were measured on the semithin sections by toluidine blue staining analysis (Figure 5). In injured and repaired sciatic nerve by cell transplantation, axons are less evenly distributed and in a smaller diameter than the normal sciatic nerve (Figure 5(a)). In quantitative analysis of multiple sections, the number of axons per 100 *μ*m × 100 *μ*m was lower in the normal sciatic nerve (39.5 ± 9.68) than for eGFP-SCs^N^ transplanted group (43.0 ± 2.16) and eGFP-SCs^I^ transplanted group (57.75 ± 8.18) (Figure 5(b)). The diameter of the axon including the myelin sheath in the eGFP-SCs^I^ group was smaller than other groups. The majority was ranged at a size of 5.0–7.0 *μ*m (mean diameter: 5.9 ± 1.2 *μ*m) but, in the eGFP-SCs^N^ group, the diameter of the axon was ranged at a size of 5.0–9.0 *μ*m (mean diameter: 6.7 ± 1.6 *μ*m), relatively bigger than that in the eGFP-SCs^I^ group (Figure 5(c)). The thickness of the myelin sheath in the eGFP-SCs^I^ group (mean thickness: 1.4 ± 0.2 *μ*m) was thinner than that in the eGFP-SCs^N^ group (mean thickness: 1.6 ± 0.5 *μ*m) but there was no statistical significance (Figure 5(d)). Overall, transplanted eGFP-SCs^N^ repaired the injury more closely to that of normal sciatic nerve than eGFP-SCs^I^ but the axon diameter and the thickness of myelin sheath in the eGFP-SCs^N^ transplanted group and eGFP-SCs^I^ transplanted group were rather variable even in the same group.

Taken together, from the in vivo transplantation experiments, we concluded that iSCs could be successfully engrafted and migrate longer along the axon in the sciatic nerve injury than nSCs. In a comparative analysis with nSCs, all the nSCs were tightly engaged in functional myelin sheath. However, only some of iSCs could differentiate into functional SC-like cells but a portion of the cells did display features of fibrocyte/fibroblast or periendoneural cells.

## 4. Discussion

We performed a comparative study to evaluate the fate of the SC-like cells differentiated from rat subcutaneous fat tissue in the injured sciatic nerve model with that of nSCs isolated from normal sciatic nerve. In this investigation, nSCs were found to be engaged only in the myelin sheath reformation at the lesion site of the injured sciatic nerve, suggesting that their SC fate is highly guaranteed in vivo. In contrast, the iSCs displayed SC traits in vivo differentiated into cells other than SCs at the lesion site, which may probably be fibrocytes/fibroblasts, endoneural cells, and perineural cells. Also, the iSCs showed much higher cell migration and cell proliferation capacity than those of nSCs. Therefore, nSCs are strictly restricted to the SC lineage even after cell expansion for several passages but iSCs, even though expressing most markers of immature SCs, are more plastic than the nSCs in vivo under the injury environment.

The subcutaneous fat tissue is composed of mainly adipose cells and small numbers of connective and endothelial cells. Adipose-derived adult stem cells have attracted intensive attention for clinical regeneration investigations [18]. Among them, very limited numbers of reports proved that adipose-derived SC-like cells have definitive capability of forming myelin structures on axon surface in in vivo environment [22, 27]. Also, how many of transplanted Schwann cell-like cells are displaying functional SC properties or choose other cell fates than SCs for the repair of injured tissue has not been investigated yet. As shown in this study, most of the induced SCs derived from the adipose tissue of eGFP-expressing transgenic rat expressed immature SCs such as A2B4 and O4 but only small portion of the induced cells did express mature marker S100*β*, which was one of the marked differences between the induced SCs and naïve ones. Much lower expression of S100*β* in the induced SCs than naïve ones suggests that induced SCs are in the more immature state. Again, much wider and longer distribution of eGFP-SCs^I^ at the injured sciatic nerve than naïve ones also suggests that their immaturity capacity is still maintained even after the transplantation, which may be important in fate plasticity. If the induced cells could be sorted to subpopulations based on S100*β* expression level or cell-proliferating capacity, the cell fate after transplantation may be estimated priorly. However, which molecular markers are representing the hierarchy of different stages of the SC differentiation needs to be further explored.

The crush-sciatic nerve injury adopted in this study may not be the best system to investigate sole effect of the transplanted SCs on the tissue repair while competing with endogenous SCs but may be suitable enough to explore their possible fate choices in the injured tissue. After the peripheral nerve injury, SCs from proximal and distal area of lesion side dedifferentiate immature SCs and proliferate, which are known to be engaged in a variety of normal and pathophysiological events such as the Wallerian degeneration, remyelination, and axonal growth, all requiring SCs' phagocytic clearance of dead cells and cell debris, myelin sheath formation, and secretion of axon guiding trophic factors [2]. Usually, the small size of peripheral nerve injury can autonomously recover to normal level [28, 29]. In our sciatic nerve injury model, which was performed by compression of both sides of sciatic nerve, endogenous repair may also be accompanied. Likely to our expectation, the crush injury resulted in a large cavity at 1 week after the injury (Figure 2(a)) but was repaired to the similar level to normal sciatic nerve at the end of 12-week experiment even in the no-cell injected group (data not shown). Again, in naïve eGFP-SCs transplanted group, all the injured area was fully filled with NF-200-expressing axons and P0-expressing myelin sheath even though only small portion of the injury site was recovered by eGFP-SCs. Therefore, under the compression injury model, how well those transplanted cells could compete with endogenous dedifferentiated SCs may not be determined and also it may not be proper to expect beneficial effect of cell transplantation. However, the cell fate determination, the characteristics of reformed myelin sheath, and the cell survival of transplanted cells would be well exploitable by tracing eGFP expression.

The capacity of the cell survival and migration within the lesion site is also crucial for the success of the cell therapy treatment. In central nervous system, the transplanted nSCs fail to migrate significantly from the implanted site and show poor long-term survival [30]. As shown in this study of sciatic nerve injury, much higher cell survival and migration of transplanted eGFP-SCs^I^ are certainly advantageous for the cell therapy over naïve ones if their cell fate after the transplantation is expectable and controllable. In this sense, molecular signatures are necessary for different stages of SC differentiation and their relationship with fate choice and plasticity under diverse injury conditions have to be further explored for successful clinical application of numerous induced and manipulated therapeutic cells.

## 5. Conclusions

In conclusion, SC-like cells induced from the nestin-expressing spheroids of adipose tissue express most markers of immature SCs but their cell fates in the sciatic nerve injury are more plastic than naïve ones. Furthermore, their survival and migration capacity are much higher than nSCs and the induced SCs are able to differentiate to perineural fibroblast-like cells without any sign of adipocyte differentiation and tumor formation. Even though induced SCs are accessible and promising cell sources for peripheral nerve injury by substituting autologous SCs, their fine control of the cell fate in vivo needs further investigation.

## Supplementary Material

Supplementary Figure 1 caption: Induction of sciatic nerve injury. A. Exposure of the right thigh's sciatic nerve by opening the fascial plane between the gluteal musculature and femoral musculature. B. The injury site after crush injury induction.

## Figures and Tables

**Table 1:** Primers used in reverse transcriptase-polymerase chain reaction (RT-PCR).

Gene	Accession number	Primer (5′-3′)	Predicted size (bp)	Annealing temperature (°C)
S100*β*	NM013191.1	5′-GAGAGAGGGTGACAAGCACAA-3′	169	52
		5′-GGCCATAAACTCCTGGAAGTC-3′		
P75	NM012610.1	5′-TGTGTGAAGAGTGCCCAGAG-3′	496	52
		5′-TCCACAGAGATGCCACTGTC-3′		
PMP22	NM017037.1	5′-TCCTCATCTGTGAGCGAATG-3′	163	52
		5′-ACAGACCAGCAAGGATTTGG-3′		
SCIP	NM138838.1	5′-ATTCCCCGGGAGAATGGACGAAAAGAGGAGAGTCC-3′	126	62
		5′-AGGTGCGAGAAGAGGCGCGGAAAGAATAAAGTGAC-3′		
Krox-20	NM053633.1	5′-AGATACCATCCCAGGCTCAGT-3′	300	56
		5′-CTCTCCGGTCATGTCAATGTT-3′		
L1	NM017345.1	5′-TGGAAGTGGAGGAAGGAGAAT-3′	202	52
		5′-AAGTGGGCATTGCAGATGTAG-3′		
ErbB2	NM017003.2	5′-AATGCCAGCCTCTCATTCCTG-3′	235	52
		5′-GACTTCGAAGCTGCAGCTCC-3′		
PDGFr-*α*a	NM012802.1	5′-CTGTAACTGGCAGGCTCGGAG-3′	334	55
		5′-GTTGTCTGCAGTACAAGTTGGCG-3′		
NSE	M11931.1	5′-TGTGGTGGAGCAGGAGAAGC-3′	565	58
		5′-GATGCATCGGGAAGGGTCAG-3′		
GAPDH	DQ403053.1	5′-ATGGGAAGCTGGTCATCAAC-3′	438	58
		5′-GGATGCAGGGATGATGTTCT-3′		
