# Supplementary figures and images for: Comparative Analysis of the Cell Fates of Induced Schwann Cells from Subcutaneous Fat Tissue and Naïve Schwann Cells in the Sciatic Nerve Injury Model
Source: Biomed Res Int. 2017 Jun 20;2017:1252851. doi: 10.1155/2017/1252851 (PMC5496110; doi:10.1155/2017/1252851)

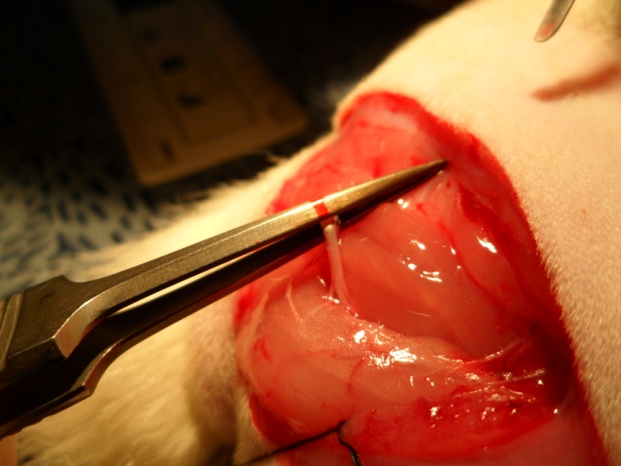

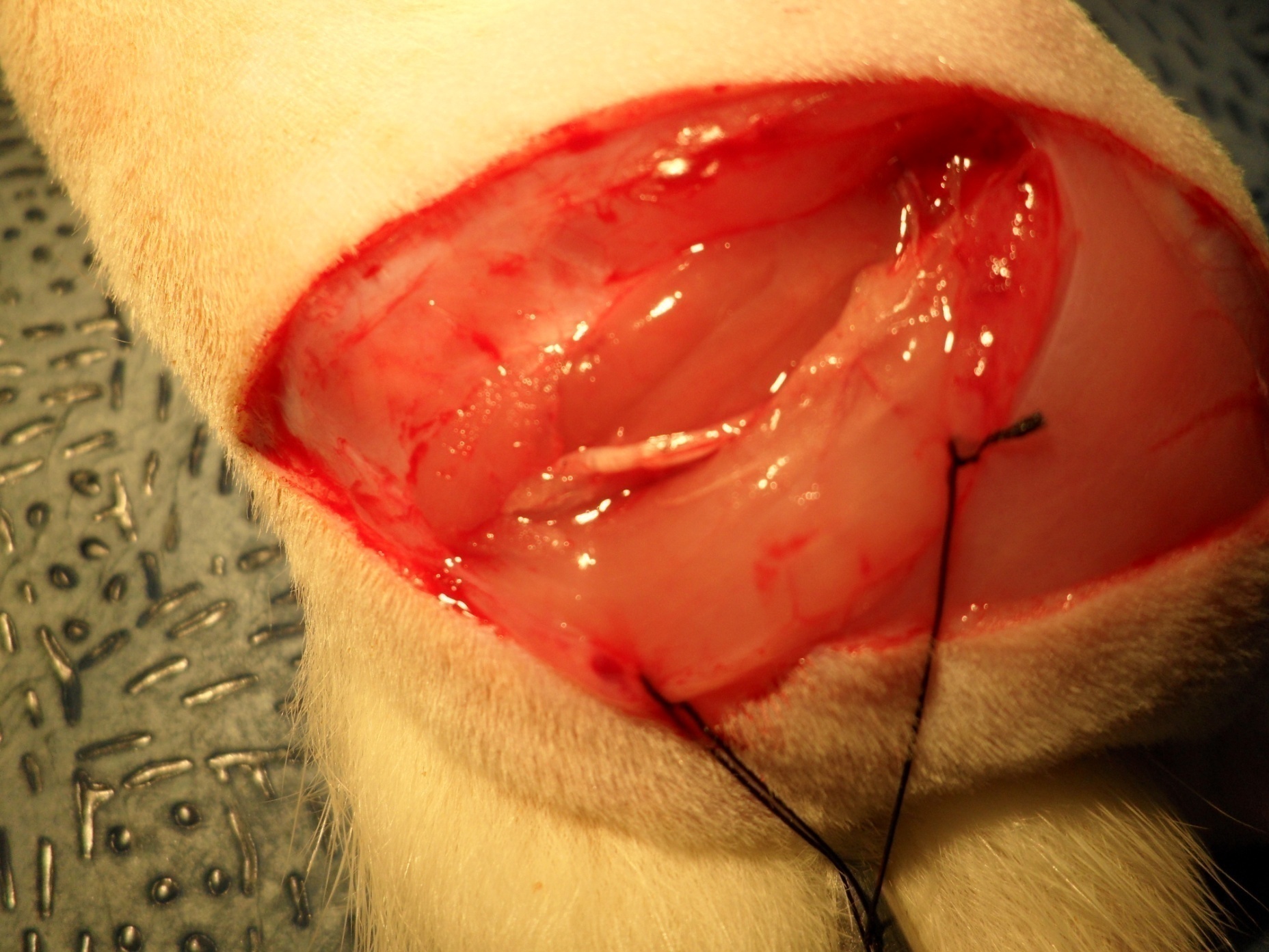


**A**

**B**

Supplemental data

**Figure 1**

Supplement: Supplementary file 1 — Supplementary Figure 1 caption: Induction of sciatic nerve injury. A. Exposure of the right thigh's sciatic nerve by opening the fascial plane between the gluteal musculature and femoral musculature. B. The injury site after crush injury induction. [file 1252851.f1.docx]
